# Multi‐Level Switching of Spin‐Torque Ferromagnetic Resonance in 2D Magnetite

**DOI:** 10.1002/advs.202401944

**Published:** 2024-05-05

**Authors:** Zhiyan Jia, Qian Chen, Wenjie Wang, Rong Sun, Zichao Li, René Hübner, Shengqiang Zhou, Miming Cai, Weiming Lv, Zhipeng Yu, Fang Zhang, Mengfan Zhao, Sen Tian, Lixuan Liu, Zhongming Zeng, Yong Jiang, Zhongchang Wang

**Affiliations:** ^1^ Institute of Quantum Materials and Devices School of Materials Science and Engineering Tiangong University Tianjin 300387 China; ^2^ International Iberian Nanotechnology Laboratory (INL) Braga 4715‐330 Portugal; ^3^ Key Laboratory of Quantum Materials and Devices of Ministry of Education School of Physics Southeast University Nanjing 211189 China; ^4^ Key Laboratory of Nanodevices and Applications Suzhou Institute of Nano‐Tech and Nano‐Bionics CAS Suzhou 215123 China; ^5^ College of Science China Agricultural University Beijing 100083 China; ^6^ Institute of Ion Beam Physics and Materials Research Helmholtz‐Zentrum Dresden‐Rossendorf Bautzner Landstrasse 400 D‐01328 Dresden Germany; ^7^ Department of Physics Beijing Normal University Beijing 100875 China; ^8^ School of Chemistry Beihang University Beijing 100191 China

**Keywords:** 2D magnetite, antiphase boundary, multi‐level resistance, spin dynamics, spin texture

## Abstract

2D magnetic materials hold substantial promise in information storage and neuromorphic device applications. However, achieving a 2D material with high Curie temperature (*T*_C_), environmental stability, and multi‐level magnetic states remains a challenge. This is particularly relevant for spintronic devices, which require multi‐level resistance states to enhance memory density and fulfil low power consumption and multi‐functionality. Here, the synthesis of 2D non‐layered triangular and hexagonal magnetite (Fe_3_O_4_) nanosheets are proposed with high *T*_C_ and environmental stability, and demonstrate that the ultrathin triangular nanosheets show broad antiphase boundaries (bAPBs) and sharp antiphase boundaries (sAPBs), which induce multiple spin precession modes and multi‐level resistance. Conversely, the hexagonal nanosheets display slip bands with sAPBs associated with pinning effects, resulting in magnetic‐field‐driven spin texture reversal reminiscent of “0” and “1” switching signals. In support of the micromagnetic simulation, direct explanation is offer to the variation in multi‐level resistance under a microwave field, which is ascribed to the multi‐spin texture magnetization structure and the randomly distributed APBs within the material. These novel 2D magnetite nanosheets with unique spin textures and spin dynamics provide an exciting platform for constructing real multi‐level storage devices catering to emerging information storage and neuromorphic computing requirements.

## Graphical abstract

The synthesis of environmentally stable and high Curie temperature 2D Fe_3_O_4_ nanosheets are reported that exhibit multi‐level resistance under the stimulation of a microwave field at room temperature. Such behavior is ascribed to the emergence of bAPBs and sAPBs in the Fe_3_O_4_ structure and morphological evolution of spin texture. The findings pave a way for synthesizing environmentally stable 2D magnetic materials at room temperature, holding substantial potential for developing practical multistage memory devices upon 2D materials.

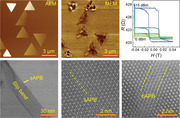

## 1 Introduction

Multi‐level magnetization switching generally bears the merits of remarkably high storage density, low power consumption, and multi‐functionality in spintronic devices, yet magnetization switching of the existing magnetic materials holds mostly a unitary pattern, necessitating structural design and development of novel magnetic materials.^[^ ^1^, ^2^, ^3^, ^4^ ^]^ 2D magnetic materials often possess the superiorities of atomic thickness, strong anisotropy, easy stacking, and high spin polarization, making them a promising solution to current challenges.^[^ ^5^, ^6^, ^7^, ^8^ ^]^ Specifically, they are expected to replace the conventional magnetic metal thin films in heterojunction spin devices by reversing their spin textures through spin current.^[^ ^9^, ^10^ ^]^ However, most of the recently investigated layered 2D magnetic materials suffer from either air sensitivity or relatively low Curie temperature (*T*_C_), posing a significant hurdle to their practical application in spintronics.^[^ ^11^, ^12^, ^13^, ^14^, ^15^ ^]^ Exploring new 2D magnetic materials and structures with both air stability and high *T*_C_ is therefore timely and ultimately important in designing multi‐level storage devices suitable for magnetic memory and neuromorphic applications.

Recently, 2D non‐layered magnetic materials,^[^ ^16^, ^17^, ^18^, ^19^ ^]^ e.g., *ε*‐Fe_2_O_3_,^[^ ^20^ ^]^ *γ*‐Fe_2_O_3_,^[^ ^21^ ^]^ Fe,^[^ ^22^ ^]^ Fe_7_Se_8_,^[^ ^23^ ^]^ CoFe_2_O_4_,^[^ ^24^ ^]^ and Fe_3_O_4_,^[^ ^25^ ^]^ were prepared experimentally, which exhibit relatively high *T*_C_ of above room temperature compared to the reported 2D layered magnetic materials.^[^ ^26^, ^27^ ^]^ Notably, 2D non‐layered magnetite (Fe_3_O_4_) was grown in a unidirectional epitaxial orientation on a substrate^[^ ^28^, ^29^ ^]^ and displays a Verwey transition that is tuneable with ionic gating.^[^ ^30^ ^]^ Albeit that Fe_3_O_4_ bulk and thin films were reported to show antiphase boundaries (APBs) and spin textures, it remains unclear whether they emerge in 2D Fe_3_O_4_ and how these structural defects can impose impact on their magnetoelectric and spin properties in reduced dimension.^[^ ^31^, ^32^, ^33^ ^]^ In addition, spin transport and spin switching properties of 2D Fe_3_O_4_ are yet to be explored despite of their crucial importance for ultrathin spintronic devices.

In this study, we conducted a controlled synthesis of 2D triangular and hexagonal Fe_3_O_4_ nanosheets using a facile chemical vapor deposition (CVD) method. Our results reveal that the triangular nanosheets exhibit broad antiphase boundaries (bAPB) and sharp antiphase boundaries (sAPB), which are strongly associated with their spin precession modes, while the hexagonal ones exhibit unique slip bands with sAPBs that pin down domain motrion. As a consequence of the APBs and spin texture in these nanosheets, we observe multi‐level resistance by modulating a magnetic field under a microwave field in the fabricated spin‐torque ferromagnetic resonance (ST‐FMR) devices upon the 2D nanosheets, which is attributed to both multi‐level magnetization switching and the induced inverse spin Hall effect. These findings highlight the potential of 2D non‐layered magnetite nanosheets as promising materials for developing next‐generation multi‐level storage devices suitable for emerging memory and neuromorphic applications.

## 2 Results and Discussion

### 2.1 Growth of 2D Fe_3_O_4_ Nanosheets

**Figure 1:**
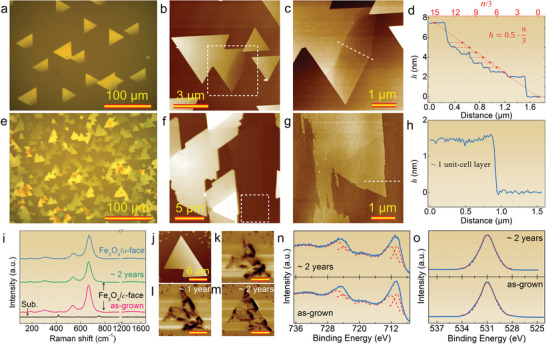
Characterization of 2D magnetite nanosheets. a,b) Optical (a) and AFM (b) images of Fe_3_O_4_ nanosheets grown on *c*‐face Al_2_O_3_. c) High‐magnification AFM image of the quadratic area marked in (b). d) Height profile acquired along the dashed line in (c) showing an average height step of ≈0.5 nm (≈1/3 unit cell). e,f) Optical (e) and AFM (f) images of Fe_3_O_4_ nanosheets grown on *a*‐face Al_2_O_3_. g) High‐magnification AFM image of the quadratic area marked in (f). h) Height profile acquired along the dashed line in (g) showing a thickness of ≈1 unit cell for the nanosheet. i) Raman spectra of the substrate, as‐grown Fe_3_O_4_ nanosheets on *c*‐ and *a*‐face Al_2_O_3_ substrates, and the corresponding Fe_3_O_4_/*c*‐face Al_2_O_3_ sample stored in air for ≈2 years. J–m) AFM (j) and MFM images of a fresh as‐grown Fe_3_O_4_ nanosheet (k) and the same sample stored for ≈1 (l) and ≈2 (m) years. n,o) High‐resolution Fe 2p (n) and O 1s (o) XPS spectra for the fresh as‐grown and 2‐year‐aged Fe_3_O_4_ sample.

2D Fe_3_O_4_ nanosheets with a thickness down to one unit‐cell layer were synthesized by the CVD method on both *c*‐ and *a*‐face Al_2_O_3_ substrates (Figure S1a, Supporting Information). As shown in **Figure** **1**a,e, the Fe_3_O_4_ nanosheets grown onto both the *c*‐ and *a*‐face Al_2_O_3_ take a triangular morphology, and the nanosheets grown on *a*‐face Al_2_O_3_ are prone to exhibit much higher nucleation density than those on *c*‐face Al_2_O_3_ (Figures S1b and Figure S2, Supporting Information), which may be related to the two‐fold lattice symmetry of the *a*‐face Al_2_O_3_.^[^ ^34^ ^]^ Further atomic force microscopy (AFM) imaging reveals that the nanosheets grown on *c*‐face Al_2_O_3_ possess a one‐step thickness of ≈0.5 nm (Figure 1b–d), which equals to 1/3‐unit cell of Fe_3_O_4_ bulk (≈1.5 nm). In contrast, thickness of the nanosheets grown on *a*‐face Al_2_O_3_ is estimated to be ≈1.5 nm (Figure 1f–h), close to one unit‐cell layer. The differences imply a strong substrate effect due to the distinct lattice mismatch between the Fe_3_O_4_ and substrates (Figure S3, Supporting Information). Nevertheless, the thick triangular nanosheets with a lamellar morphology are grown in four vertical growth modes: helical, epitaxial, misaligned, and overlapped stacking pattern. The helical stacking model involves screw dislocation‐driven growth as the primary formation factor.^[^ ^35^, ^36^, ^37^, ^38^ ^]^ On the other hand, the epitaxial growth corresponds to a conventional vertical stacking model. The misaligned and overlapped stacking patterns are created by the densely packed multiple epitaxial growth patterns (Figure S1c–n, Supporting Information).

Figure 1i shows Raman spectra of the Fe_3_O_4_ nanosheets grown on both *c*‐ and *a*‐face Al_2_O_3_, where four peaks can be identified at ≈188.4, ≈304.1, ≈536.7, and ≈666.4 cm^−1^, which point to the *T*_2g_(1), *E*_g_, *T*_2g_(2), and *A*_1g_ mode of Fe_3_O_4_, respectively,^[^ ^25^ ^]^ implying identical chemical composition of the nanosheets grown on the two substrates. The intensity of Raman peaks increases gradually with thickness of the nanosheets, yet no clear shift in peak position is visible, which is attributed to the absence of significant internal strain in the non‐layered structure of Fe_3_O_4_ grown on sapphire (Figure S4, Supporting Information).^[^ ^25^, ^39^ ^]^ Note that we also conducted Raman analysis for the nanosheets stored in a clean room for 2 years, and found that they show the same Raman signals as the fresh as‐grown samples (Figure 1i), indicative of excellent air stability of the nanosheets. Moreover, the freshly prepared samples exhibit nearly identical X‐ray photoelectron spectroscopy (XPS) peaks to those stored for a duration of 2 years (Figure 1n,o), particularly in the aspects of peak position and full width at half maximum (FWHM) (Table S1, Supporting Information), indicating that the chemical states of Fe and O are environmentally stable in the nanosheets. Furthermore, the nanosheets also show stable spin textures at room temperature for 2 years (Figure 1j–m), further verifying the magnetic and environmental stability of the samples.

To investigate the magnetic properties of the nanosheets, we conducted zero‐field‐cooling (ZFC) and field‐cooling (FC) measurements (Figure S5a, Supporting Information) in a temperature range of 5–400 K under out‐of‐plane (OOP) and in‐plane (IP) magnetic fields of 500 Oe. The as‐grown sample shows a typical first‐order metal‐insulator Verwey transition at a Verwey transition temperature (*T*_V_) of ≈122 K (Figure S5a, Supporting Information), at which it transforms from the cubic *Fd* $\bar{3}$ *m* to the monoclinic *Cc* structure.^[^ ^40^ ^]^ The magnetic field exhibits a steeper slope of hysteresis (*M*‐*H*) loops along the IP than the OOP direction under a near‐zero magnetic field for the as‐grown nanosheets (Figure S5b,c, Supporting Information), indicative of magnetic anisotropy for the 2D nanosheets.^[^ ^41^ ^]^ Furthermore, Figure S3d (Supporting Information) presents the temperature‐dependent remnant magnetization (*M*_R_), saturation magnetization (*M*_S_), and coercivity (*H*_C_) for the nanosheets with magnetic fields along the IP and OOP directions. Both the *H*_C_ and *M*_R_ undergo remarkable changes as temperature decreases below *T*_V_. In addition, we compared *H*_C_ with its reported values in literature and found them to be very similar as detailed in Table S2 (Supporting Information).^[^ ^29^, ^39^ ^]^ By analyzing the squareness‐temperature curves under both in‐plane and out‐of‐plane magnetic fields (Figure S6, Supporting Information), we have determined that the *M*‐*H* hysteresis curve exhibits a more rectangular shape for the in‐plane field orientation, indicating that the easy magnetization axis of Fe_3_O_4_ lies in in‐plane direction.^[^ ^42^, ^43^ ^]^ It is worthy of noting that the thickness of nanosheets is predominantly distributed within the range of 0 and 25 nm. A Gaussian fit applied to the frequency plot of the thickness distribution reveals a central value at 13.4 nm (Figure S7, Supporting Information).

To investigate the phase transition temperature (*T*_C_) of the 2D nanosheets, we measured *M*‐*H* loops over a wide temperature range from 300 K to 985 K under an IP magnetic field for a fresh sample (Figure S5e, Supporting Information). The nanosheets maintain ferrimagnetism even at high temperature, as revealed in the high‐temperature *M*‐*H* loops and the ZFC‐FC curves (Figure S5e,f, Supporting Information). The *T*_C_ is estimated to be above 850 K from the minimum position of the dM/dT versus temperature (Figure S5f, Supporting Information), which differs from that of the ultrathin nanosheets owing to the thickness effect.^[^ ^29^ ^]^

### 2.2 Atomic‐Scale Structure and Antiphase Boundary

**Figure 2:**
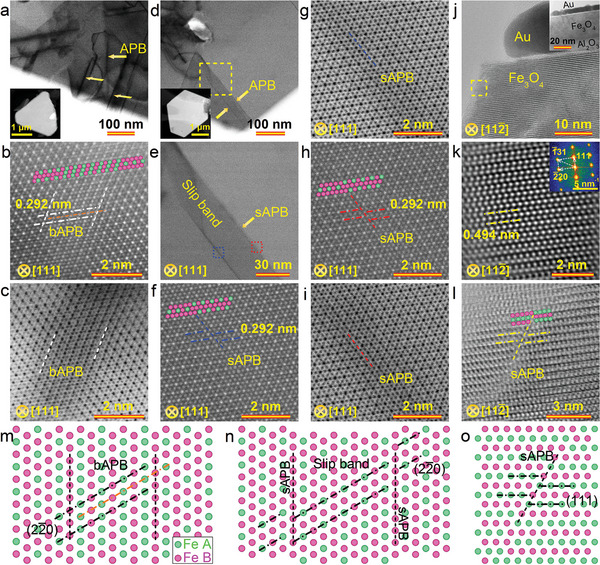
Atomic‐scale analysis of the nanosheets. a) BF STEM image of an edge of a triangular Fe_3_O_4_ nanosheet. The inset shows a STEM image of the triangular nanosheet. The arrows mark the APB locations. b,c) HAADF (b) and BF (c) STEM images taken at an APB area. d) BF STEM image of a corner of a hexagonal Fe_3_O_4_ nanosheet. The inset shows a STEM image of the hexagonal nanosheet. e) Amplified BF STEM image of a slip band region marked as a square in (d). f–i) HAADF (f,h) and BF (g,i) STEM images of an APB taken from the quadratic area marked by a blue frame on the left side of the slip band (f,g) and a red frame on the right side of the slip band (h,i) in (e). j,k) TEM (j) and HRTEM (k) images of a cross‐sectional sample showing a lattice plane spacing of 0.494 nm, which corresponds to the (111) plane. The inset in (j) shows an overview of the cross‐sectional lamella, and the inset in (k) gives a fast Fourier transform pattern of Fe_3_O_4_. l) HRTEM image of an sAPB taken from the quadratic area marked by a dashed frame in (j). Cyan and pink balls in the STEM images represent Fe A‐ and B‐sites, respectively. m–o) Atomic models illustrating the arrangement of Fe A‐ and B‐site atoms in the bAPB (left), slip band (center, including sAPB), and sAPB (right) area. Only Fe atoms are presented for clarity.

Magnetite exhibits a cubic spinel‐type structure in which Fe^3+^ and Fe^2+^ occupy the tetrahedrally coordinated A and octahedrally coordinated B cation sites, respectively.^[^ ^40^ ^]^ **Figure** **2**a–i shows scanning transmission electron microscopy (STEM) images of a triangular and hexagonal nanosheet, both of which show honeycomb lattice fringes, indicative of single crystallinity of high quality for the nanosheets. The disparity in morphology primarily arises from the variation in elemental precursors of Fe and O elements.^[^ ^44^, ^45^ ^]^ The observed interplanar distance of ≈0.292 nm between lattice fringes corresponds to (2$\bar{2}$0) lattice planes of the magnetite. Further spectrum imaging analysis based on energy‐dispersive X‐ray spectroscopy (EDX) reveals a uniform distribution of Fe and O in the as‐grown nanosheets (Figure S8, Supporting Information). Interestingly, high‐angle annular dark‐field (HAADF) STEM images reveal the presence of antiphase boundaries (APBs) in both the triangular (Figure 2b) and hexagonal (Figure 2F,H) nanosheets, as also confirmed by the corresponding bright‐field STEM (BF STEM) images (Figure 2a,c–e,g,i).^[^ ^31^, ^32^, ^46^ ^]^ The triangular nanosheet exhibits abundant broad APBs (bAPBs), which are observed as transition bands (Figure 2a) where Fe A‐ and B‐sites interchange positions (marked by parallel black and blue lines in Figure 2b,c). Nanoscale slip bands can also be identified in the hexagonal nanosheet (Figure 2d,e), accompanied by sharp APBs (sAPBs) on either sides. The Fe A‐ and B‐sites are regularly interchanged across the black and red lines marked in the HAADF and BF STEM images (Figure 2f–i).

Figure 2j,k shows cross‐sectional TEM and high‐resolution TEM (HRTEM) images taken along the [11$\bar{2}$] zone axis for a triangular Fe_3_O_4_ nanosheet deposited on *c*‐face Al_2_O_3_ (Figure S9, Supporting Information). The interplanar spacing of the (111) plane is estimated to be 0.494 nm, consistent with the findings in Figure 1d revealing a thickness of ≈0.5 nm (1/3 unit cell) for the nanosheet. Fast Fourier transform (FFT) analysis of the Fe_3_O_4_ region further reveals one set of quadrilateral spots (Figure 2k, inset), confirming single crystallinity of the 2D nanosheets. A sAPB can be clearly observed (Figure 2l), along which the Fe A‐ and B‐sites are swapped in the (111) plane. Figure 2m–O shows atomic models of the Fe A‐ and B‐site substitution at the bAPB, slip band, and sAPB, in which the Fe A‐ and B‐sites at the bAPB are interchanged gradually, while those at the sAPB are straightforward replaced on each side of a boundary.

**Figure 3:**
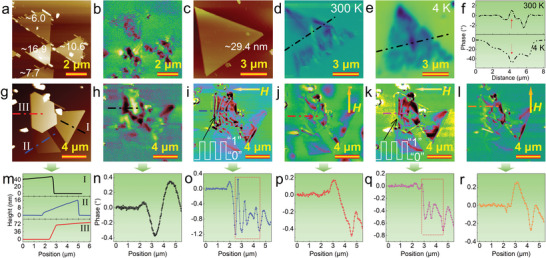
Spin texture modulation. a,b) AFM (a) and MFM (b) images of several triangular Fe_3_O_4_ nanosheets with different thickness. The numbers given in (a) indicate the corresponding thickness of the nanosheets in nanometers. c–e) AFM (c) and MFM (d,e) image of a triangular Fe_3_O_4_ nanosheet measured at 300 K and 4 K. f) MFM phase angle distribution along the dashed lines in (d) and (e). g,h) AFM (g) and MFM (h) images of three as‐grown triangular and hexagonal nanosheets. i,j) MFM images taken in the corresponding area in (g) after annealing at 603 K under a magnetic field of 1 T for two applied magnetic field directions. The applied magnetic field, which is indicated by an arrow, is perpendicular (i) and parallel (j) to an edge of the hexagonal nanosheet. The line chart displayed at the bottom left accurately represents the oscillation of the spin texture signal, resembling a seamless switching between the “0” and “1” signals. k,l) MFM images measured after repeated annealing at 603 K under a magnetic field of 1 T. The arrows point to the direction of the applied magnetic field. m–r) AFM height and MFM phase angle profile lines achieved along the dashed lines in the respective AFM and MFM images.

To elucidate the influence of thickness and temperature on the spin textures, we conducted magnetic force microscopy (MFM) characterization of the 2D nanosheets with different thickness (**Figure** **3**a,b). The spin texture signals are robust at room temperature and intensify with the increase in sample thickness, as confirmed by the MFM analysis of other samples with a thickness range of 5.15–93.87 nm (Figure S10, Supporting Information). The nanosheets retain a multi‐domain structure even at a thickness of ≈5.15 nm, while those below 5 nm exhibit ambiguous domains.^[^ ^47^, ^48^ ^]^ The phase angle of the spin texture increases by over 20 times as temperature drops from 300 to 4 K, indicating that spin texture is rather sensitive to temperature for the 2D nanosheets.^[^ ^49^ ^]^ Furthermore, significant flip in spin texture was observed at low temperatures across the nanosheets with different thicknesses (Figure S11, Supporting Information).

Modulating spin textures is crucial for advancing the applications of magnetic materials. To this end, we annealed an as‐grown sample at 603 K under a magnetic field of 1 T, and found that the shape of the spin textures is tunable by rotating in‐plane magnetic field (Figure 3g–r). When the in‐plane magnetic field turns perpendicular to a boundary of the nanosheet, there emerge the sharp‐shaped magnetic striped labyrinth domains at the surface of the hexagonal nanosheet (Figure 3i,o), which are not observed in the pre‐annealed sample (Figure 3h,n). These domains likely arise from the competition between magnetic field driving and slip bands (containing sAPBs) pinning effects within the hexagonal nanosheet. However, such domains can be erased when the magnetic field is rotated in‐plane by 90° during annealing. In this way, these stripes could be repeatedly erased and written, which corresponds to the switching signal of “0” and “1”, respectively. Nearly all spin textures could be erased during annealing under an OOP magnetic field (Figure S12, Supporting Information), where magnetic moments are nearly aligned uniformly along one direction, suggesting the feasibility of using 2D Fe_3_O_4_ nanosheets for encrypted information storage.

### 2.3 Magnetoelectric Transport, Spin Dynamics, and Spin Transport

**Figure 4:**
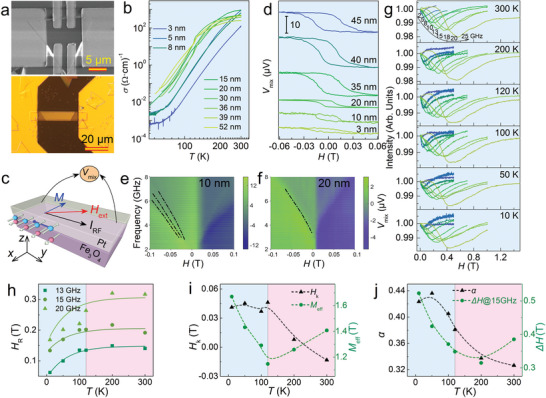
Thickness‐dependent electrical transport, FMR, and ST‐FMR performance. a) SEM (upper) and optical microscopy (lower) images of the electrical transport and ST‐FMR devices, respectively. b) Temperature‐dependent electrical conductivity (*σ*) for Fe_3_O_4_ nanosheets with different thickness. c) Sketch of the Fe_3_O_4_/Pt (4 nm) ST‐FMR device. d) Room‐temperature ST‐FMR signal of the Fe_3_O_4_/Pt (4 nm) ST‐FMR devices with Fe_3_O_4_ nanosheets of different thickness. e,f) Resonance peak mapping images of Fe_3_O_4_ nanosheets with a thickness of 10 and 20 nm. g) Magnetic field dependence of the FMR signals for the Fe_3_O_4_ nanosheets measured under an in‐plane magnetic field at different temperature. h–j) Temperature‐dependent resonance field (h), in‐plane anisotropic field and effective magnetization (i), Gilbert damping and resonance linewidth (j) extracted from the FMR spectra in (g).

To explore magnetoelectric transport of the 2D nanosheets, we fabricated Hall and coplanar waveguide devices using the as‐grown triangular nanosheets (**Figure** **4**a). Figure 4b presents the temperature‐dependent conductivity (*σ*) of the devices based on the nanosheets of different thickness, where an abrupt decrease in *σ* emerges at ≈120 K for thick devices due to the classical metal‐insulator Verwey transition.^[^ ^50^, ^51^ ^]^ However, for the samples with a thickness of no more than 15 nm, the transition temperature decreases slightly and transition trend broadens, which can be ascribed to the stress effect triggered by the lattice mismatch between the nanosheets and the *c*‐face Al_2_O_3_ substrate. Such effect turns out more severe for thinner samples.^[^ ^52^ ^]^

To gain insights into the impact of thickness on spin dynamics of Fe_3_O_4_ nanosheets, we first conducted spin‐torque ferromagnetic resonance (ST‐FMR) measurements by depositing a Pt thin film (4 nm) onto the Fe_3_O_4_ nanosheets to form a coplanar waveguide heterostructure (Figure 4c).^[^ ^53^ ^]^ The RF current (*I*_RF_) applied to the device triggers a time‐varying driving force on the magnetization and subsequently the precession of magnetic moments. The spin current polarized along the magnetization direction backflows from Pt to Fe_3_O_4_ at the heterointerface, resulting in a resistance oscillation ∆*R*, which may be attributed to the spin Hall magnetoresistance (SMR) effect^[^ ^54^, ^55^ ^]^ and magnetic proximity effect.^[^ ^56^ ^]^ The rectified voltage between the oscillated resistance and microwave signal can then be detected. Additionally, the spin pumping and inverse spin Hall effect can also induce DC output voltage.^[^ ^57^ ^]^ Magnetization reversal and precession can be achieved by recording the effective ST‐FMR signal (*V*_mix_) under different microwave and magnetic field conditions. The FMR signal formed by the precession of magnetic moments usually appears at high magnetic field region when microwave frequency is high, while the signal at low magnetic field region is dominated by magnetization reversal.

Figure 4d illustrates the *V*_mix_ of Fe_3_O_4_/Pt for various Fe_3_O_4_ thicknesses recorded during the field sweeping process under high‐frequency (*f* = 24 GHz) microwave conditions. The observed hysteresis loops in the low field region reflect the magnetization reversal process for the Fe_3_O_4_. This reversal process exhibits a slow response to the increasing magnetic field, suggesting the presence of a multi‐domain reversal process, magnetic anisotropy, and non‐uniform internal field (e.g., demagnetizing field distribution) in the Fe_3_O_4_ nanosheets. To shed light on the spin dynamics, we further investigated the precession dynamics of magnetic moments at different frequency. The *V*_mix_ is not detectable when the Fe_3_O_4_ nanosheet is ≈3 nm thick, and three dispersion relations are unexpectedly observed in a 10‐nm‐thick nanosheet, indicating multiple non‐uniform precession modes (or spin wave modes) in this structure (Figure 4d and Figure S13c,d, Supporting Information), which might be attributed to the unique bAPBs and sAPBs in the triangular Fe_3_O_4_ nanosheets. However, the linewidth of the resonance peak widens and the FMR response disappears when the nanosheet turns thick. The multiple precession modes are not clearly detectable in thick Fe_3_O_4_ nanosheets (Figure 4f; Figure S13, Supporting Information), which is ascribed to the mode superposition caused by the non‐uniform spin dynamics in thick Fe_3_O_4_.

Apart from the ST‐FMR, we also conducted broadband FMR characterization of the as‐grown Fe_3_O_4_ nanosheets using a vector network analyzer. Figure 4g shows the FMR spectra acquired at frequencies of 2–25 GHz from 300 K to 10 K. Through fitting the spectrum with a combination of symmetric and asymmetric Lorentzian functions, we extracted the frequency, temperature‐dependent resonance field (*H*_R_), and resonance linewidth (*∆H*) (Figure S14, Supporting Information). Figure 4h presents the variation of *H*_R_ with temperature, which can be fitted by the expression *H*_R_ $\propto e^{\frac{T}{T_{v}/2}}$. This variation describes the effect of Verwey transition on spin dynamics. We further analyzed the frequency dependence of the resonance field according to Kittel's equation^[^ ^58^ ^]^

(1) $${\left(\frac{2\pi f}{\gamma }\right)}^{2}=\left(H_{R}+H_{k}\right)\;\times \left(H_{R}+H_{k}+4\pi M_{\mathrm{eff}}\right)$$

where *H*_k_ is the in‐plane magnetic anisotropy field and *M*_eff_ is the effective magnetization describing the difference between saturation magnetization and surface anisotropy. Figure 4i presents the extracted *H*_k_ and *M*_eff_ data, where *H*_k_ keeps almost stable for *T* < *T*_V_, yet decreases obviously with increasing temperature for *T* > *T*_V_. Conversely, the temperature dependence of *M*_eff_ shows an opposite trend on both sides of *T*v due to the Verwey phase transition.^[^ ^59^ ^]^

Figure 4j shows the variation of *∆H* with temperature at 15 GHz. Accordingly, the Gilbert damping (*α*) can be obtained by fitting the frequency dependence of *∆H* ^[^ ^60^ ^]^

(2) $$\Delta H\;=\Delta H_{0}+\frac{4\pi \alpha }{\gamma }f$$

where Δ*H*_0_ represents the inhomogeneous broadening of the linewidth, meaning that a value closer to zero denotes a higher quality of the nanosheet. The *α* decreases with temperature, which is evident when the temperature is around *T*_V_. The *α* of our samples is larger than that of the Fe_3_O_4_ films reported previously, which might be ascribed to the obvious spin relaxation, multi‐domain states, magnetic anisotropy, and non‐uniform demagnetizing field distribution in the Fe_3_O_4_ nanosheets triggered by the APBs.^[^ ^61^ ^]^ The findings demonstrate that 2D magnetite nanosheets are applicable for a wide range of magnetic and spintronic devices.

**Figure 5:**
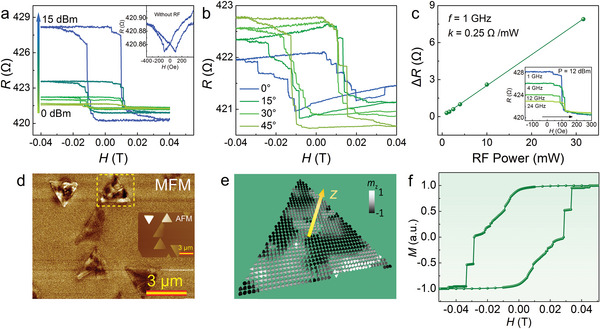
Multi‐level‐magnetization‐switching‐related spin transport. a) Magnetic‐field‐dependent resistance of the device under a microwave field of different power intensities at a frequency of 1 GHz. The inset shows the magnetic resistance of the device measured in the absence of the microwave field. b) Magnetic resistance of the device measured at different magnetic field orientations. The frequency and power of the microwave field are set to 10 GHz and 12 dBm, respectively. c) Variation in resistance of the device under microwave power. The inset gives the resistance variation at different frequencies of the microwave field. d) MFM image adopted for the micromagnetic simulation. e) Relaxation of the simulation material under an internal field. The material is chosen from the MFM image as marked by a dashed frame in (d). f) Simulated hysteresis loop of the material shown in (e).

**Figure** **5**a shows the multi‐level resistance of a Fe_3_O_4_ nanosheet with a thickness of ≈58 nm, which contravenes the conventional magnetic‐field‐dependent resistance displaying a single flip mode.^[^ ^62^ ^]^ This multi‐level resistance can be modulated by both the intensity of the microwave field (Figure 5a) and the direction of the magnetic field (Figure 5b). Moreover, the resistance of the device is tunable by the microwave power (Figure 5c), which may be triggered by the spin transport accompanied with multi‐level magnetization switching. Such multi‐level switching can only be observed under microwave excitation, i.e., it disappears once the microwave is switched off (Figure 5a, inset), indicating that it is a spin‐current‐related effect rather than a magnetic proximity effect. The underlying physical mechanism may be that the microwave excitation drives magnetic moments to proceed, which generates spin current whose polarization direction is corelated with the magnetization orientation of Fe_3_O_4_. This spin current flows through the Fe_3_O_4_/Pt interface, triggering an inverse spin Hall effect in Pt,^[^ ^48^ ^]^ which transforms into charge current that superimposes on the test current of the magnetoresistance (MR). Such a switching process is of multi‐level nature owing to the multi‐domain magnetic structure of Fe_3_O_4_, and the spin currents generated by the precession in different magnetic domain regions bear various polarization directions. The resulting charge currents thus hold different orientations, which superimpose on each other, leading to variations in the overall electrical signal with the multi‐stage magnetization switching process.

To elucidate the mechanism underlying multi‐level magnetization switching, we conducted micromagnetic simulations using the observed MFM image (Figure 5d) and employed the Object Oriented MicroMagnetic Framework (OOMMF) software to simulate the hysteresis loop.^[^ ^63^ ^]^ The magnetization configuration is first read out based on intensity of the MFM image. Figure 5e shows a stable magnetic state of the nanosheet in Figure 5d (marked by a dashed white frame) after relaxation under internal fields of the material including anisotropic, demagnetizing and exchange field. By considering the multi‐spin texture, magnetization structure, and randomly distributed APBs within the nanosheet, our simulated hysteresis loop (Figure 5f) reveals a distinct multi‐level state of magnetization switching. The multi‐level switching behavior varies remarkably for the materials with different spin textures, yet turns invisible for those with a uniform magnetization orientation (Figure S15, Supporting Information). The significance of such spin transport associated with multi‐level magnetization switching lies in its potential application toward the development of advanced multi‐level storage devices.

## 3 Conclusion

Seeking 2D magnetic materials with multi‐level resistance states is of ultimate significance for their prospective applications in the field of information storage. In order to investigate the magnetic switching behavior of non‐layered Fe_3_O_4_ in reduced dimension, we successfully synthesized triangular and hexagonal Fe_3_O_4_ nanosheets with varying thicknesses by the CVD method. Our findings demonstrate that due to the presence of unique bAPBs and sAPBs within these nanosheets, the FMR device upon the triangular Fe_3_O_4_ shows a wide *∆H* and a large *α* due to its large spin relaxation, internal multi‐domain states, magnetic anisotropy, and non‐uniform demagnetizing field distribution. Further ST‐FMR tests conducted on the triangular Fe_3_O_4_/Pt structures reveal a hysteresis loop in the *V*_mix_ versus magnetic field, indicating the presence of a multi‐domain reversal process and a non‐uniform internal field within the 2D Fe_3_O_4_. Unlike ultra‐thin nanosheet devices, we identify at least three dispersion relations in the ≈10‐nm ST‐FMR device suggesting multiple non‐uniform precession modes or spin wave modes are present. In contrast, the ≈58 nm device exhibits multi‐level resistance as the magnetic field varies under a microwave field, which is mainly induced by multi‐level magnetization switching, as further confirmed by micromagnetic simulations. This work opens up a new avenue in applying conventional magnetite with both air stability and high *T*_C_ in reduced dimension as an alternative for next‐generation ultrathin multi‐level magnetic information memory and neuromorphic devices.

## 4 Experimental Section

### Growth of 2D Fe_3_O_4_ Nanosheets

2D Fe_3_O_4_ nanosheets were synthesized on *a*‐face and *c*‐face Al_2_O_3_ substrates via chemical vapor deposition (CVD). A corundum boat loaded with mixed powder of Fe (0.09 g, 99.9%, Alfa Aesar) and NaCl (0.03 g, 99.99%, Alfa Aesar) was placed at the center of the furnace zone, and the clean Al_2_O_3_ substrate facing down was used as a cover on the corundum boat. The CVD system was evacuated and flushed with high‐purity Ar gas at a flow rate of 200 sccm for 10 min to eliminate air and other impurities prior to heating. Then, the furnace zone was heated to 800 °C in 30 min and maintained at 800 °C for another 30 min. Meanwhile, Ar gas at a flow rate of 30 sccm was introduced into the CVD system as carrier gas under atmospheric pressure, followed by natural cooling to room temperature to achieve 2D Fe_3_O_4_ nanosheets.

### Structural Characterization

Optical microscopy (OM) imaging was performed using an optical microscope (PSM‐1000, Moti). AFM and MFM images were collected using an atomic/magnetic force microscope (Dimension Icon, Bruker). Raman spectra and mapping were obtained using a confocal Raman microscope (Alpha 300R, WITec) with a 532‐nm laser, which was generated using a handheld optical power meter console (PM100D, Thorlabs) with a laser spot diameter of 1–2 µm. XPS measurements were performed using a spectrometer (ESCALAB 250Xi, Thermo Scientific) with Al *Kα* X‐ray source (1489.6 eV). SEM images were taken using a field‐emission scanning electron microscope (Quanta 650FEG, FEI). The STEM imaging was performed using a double‐corrected transmission electron microscope (Titan G2 Cubed Themis, FEI) operated at 200 kV with a probe convergence angle of 21.4 mrad. To characterize the chemical composition, spectrum imaging analysis based on the energy‐dispersive X‐ray spectroscopy (EDX) was conducted using a Super‐X system (integrates four windowless SDD X‐ray detectors) mounted on the STEM. TEM specimens were prepared by the standard wet transfer method.^[^ ^64^ ^]^ Cross‐sectional TEM lamellae were prepared by in situ lift‐out using a standard Ga FIB (Helios 5 CX, Thermo Fisher). To avoid charging, a gold layer was deposited onto the sample before the FIB experiments. The bright‐field and high‐resolution TEM (HRTEM) images were taken by the double‐corrected Titan 80–300 microscope (FEI) operated at an accelerating voltage of 300 kV. MFM images were obtained using a variable temperature system (attoMFM, Attocube). The annealing process under magnetic field was carried out by a commercial magnetic annealing system (Matr 2000, Magnetic Solutions).

### Magnetic Property, Device Fabrication, and Magnetoelectric Transport

Magnetic properties were measured using a superconducting quantum interference device (SQUID, MPMS3, Quantum Design) equipped with both low‐ and high‐temperature options. FMR signals were collected using a physical property measurement system (PPMS, Dynacool, Quantum Design) equipped with ferromagnetic resonance rod (FMR, MutiFields Tech.), source meters (Keithley 6221, 2182A, and 6517B), and lock‐in amplifier (Stanford, SR 830).

The Hall devices were fabricated using standard photolithography techniques, assisted by a direct‐write laser (DWL 2000, Heidelberg Instruments), an electron‐beam lithography tool (EBL, eLINE Plus, Raith), and a photoresist coater (Gamma Cluster, Karls SUSS MicroTec). Ti/Au (10 nm/60 nm) layers were then deposited onto the substrate by a conventional lift‐off process using the electron beam evaporation (EBE) with a base pressure of 3 × 10^−6^ Torr and a deposition rate of 1.0 Å/s. To pattern the magnetite into microstrips for coplanar waveguide device fabrication and high‐frequency measurements, metal deposition, exposure, and etching technologies were employed. Prior to patterning, the magnetite was covered with a Pt layer of 4 nm. Ground‐signal‐ground‐type electrical leads made of Ti/Au (10 nm/60 nm) were fabricated via optical lithography. The center and outer arms of the electrical leads had a width of 60 and 200 µm, respectively, which were separated by a gap of 20 µm. The electric leads had a nominal characteristic impedance of 50 Ω. The transport measurements were performed using the PPMS.

The spin dynamics were investigated using a homemade ST‐FMR system. An alternating current *I*_RF_ with a fixed frequency *f* was applied through the device during the measurements. An external magnetic field was exerted onto the film plane with an angle *φ* relative to the long axis of the microstrip. The output power of the microwave signal generator was set at 15 dBm. All measurements were conducted at room temperature.

## Conflict of Interest

The authors declare no conflict of interest.

## Author Contributions

Z.J., Q.C., and W.W. contributed equally to this work. Z.C.W. and Y.J. initiated and managed the project. Z.Y.J., W.J.W., F.Z., M.F.Z., and S.T. synthesized the samples and performed AFM, Raman, XPS, MFM, and VSM measurements. M.M.C. completed MFM (4 K) measurements. R.S. prepared TEM samples and conducted structural characterization. R.H. performed TEM analysis. Z.Y.J., Z.C.L., S.Q.Z., and L.X.L. fabricated the Hall devices and performed electrical measurements. Q.C., W.M.L., Z.P.Y., and Z.M.Z. fabricated the ST‐FMR devices and performed the FMR property tests. Q.C. performed micromagnetic simulations. Z.Y J., Q.C., and Z.C.W. analyzed the data and wrote the manuscript. All authors discussed the results and directed the entire study. Z.Y.J., Q.C., and W.J.W contributed equally to this work.

## Supporting information

Supporting Information

## Data Availability

The data that support the findings of this study are available from the corresponding author upon reasonable request.
