# Supplementary material for: Multi‐Level Switching of Spin‐Torque Ferromagnetic Resonance in 2D Magnetite
Source: Adv Sci (Weinh). 2024 May 5;11(26):2401944. doi: 10.1002/advs.202401944 (PMC11234467; doi:10.1002/advs.202401944)
Supplement: Supplementary file 1 — Supporting Information [file ADVS-11-2401944-s001.pdf]

ADVANCED SCIENCE Open Access

Supporting Information

for *Adv. Sci.*, DOI 10.1002/advs.202401944

Multi-Level Switching of Spin-Torque Ferromagnetic Resonance in 2D Magnetite

Zhiyan Jia, Qian Chen, Wenjie Wang, Rong Sun, Zichao Li, René Hübner, Shengqiang Zhou, Miming Cai, Weiming Lv, Zhipeng Yu, Fang Zhang, Mengfan Zhao, Sen Tian, Lixuan Liu*, Zhongming Zeng, Yong Jiang* and Zhongchang Wang**

Supporting Information

Multi-level Switching of Spin-torque Ferromagnetic Resonance in Two-dimensional Magnetite

Zhiyan Jia[#], Qian Chen[#], Wenjie Wang[#], Rong Sun[*], Zichao Li, René Hübner, Shengqiang Zhou, Miming Cai, Weiming Lv, Zhipeng Yu, Fang Zhang, Mengfan Zhao, Sen Tian, Lixuan Liu[*], Zhongming Zeng, Yong Jiang[*] and Zhongchang Wang[*]

Table S1. Fitting data of the chemical states of Fe 2p and O 1s for the as-grown Fe_3O_4 sample and the same sample after aging for two years. All peaks are fitted by taking the Gauss-Lorentz (ratio: 40%) combination formula.

Storage time	Fe^{2+} $2p_{3/2}$ (eV)		Fe^{3+} $2p_{3/2}$ (eV)		Fe^{2+} $2p_{1/2}$ (eV)		Fe^{3+} $2p_{1/2}$ (eV)		O 1s (eV)	
	Position	FWHM	Position	FWHM	Position	FWHM	Position	FWHM	Position	FWHM
as-grown	710.40	2.27	711.77	3.97	723.67	2.93	725.28	4.10	530.99	2.12
~ 2 years	710.43	2.07	711.97	3.67	723.59	2.53	725.35	3.85	530.99	2.10

Table S2. Coercivity and thickness of Fe_3O_4 samples synthesized by different methods.

Sample	Method	Thickness (nm)	H_C (Oe)	Direction	T (K)	Ref.
Fe_3O_4 nanosheets	CVD	10-25	0.014<br>0.033	IP<br>OOP	300	This work
Fe_3O_4 films	CVD	16	~ 0.04	IP	300	[1]
		4	~ 0.03	IP		
Fe_3O_4 nanosheets	CVD	15.8	0.003	IP	300	[2]
		73	0.015	IP	300	

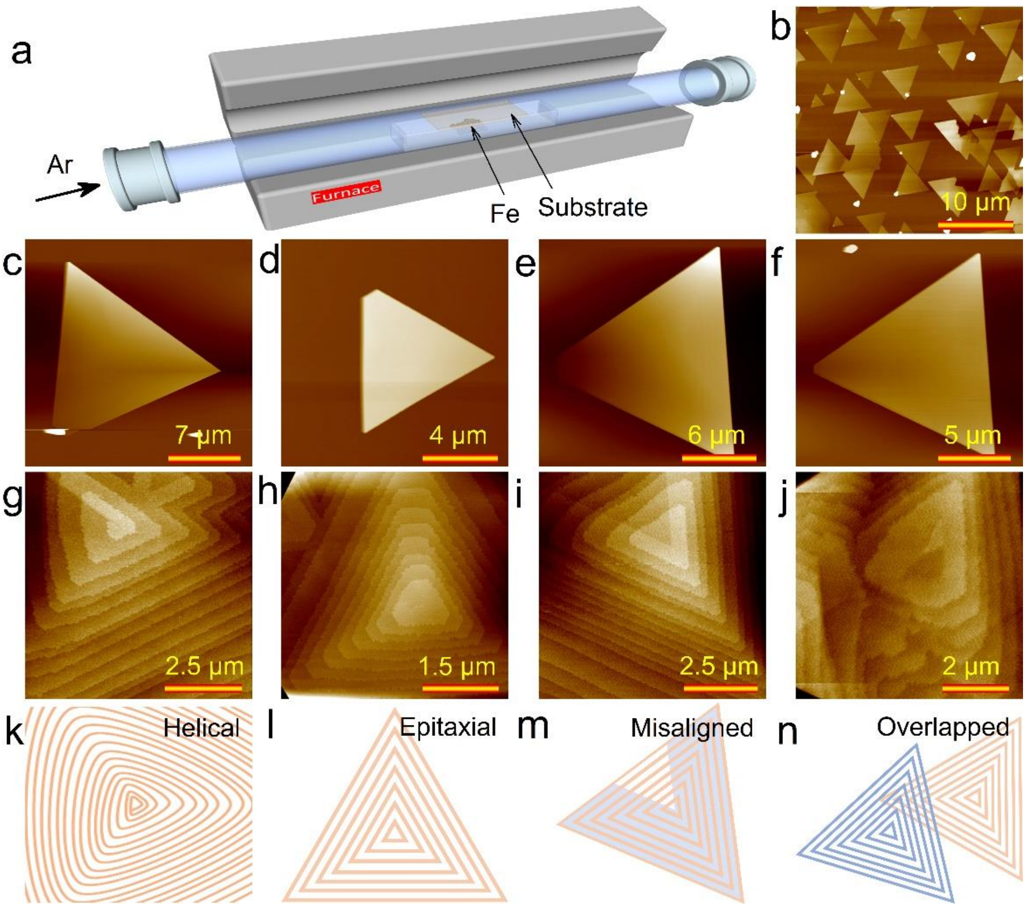

Figure S1. The CVD system and growth mode of Fe_3O_4. a) Sketch of the CVD system. b) AFM image of the fresh Fe_3O_4 nanosheets. c–f) AFM images of four different triangular Fe_3O_4 nanosheets with large thickness. (g–f) Surface profile images of the corresponding four Fe_3O_4 nanosheets shown above in (c–f). k–n) Diagram of the four stacking growth modes obtained from their respective surface profile images given in (g-j).

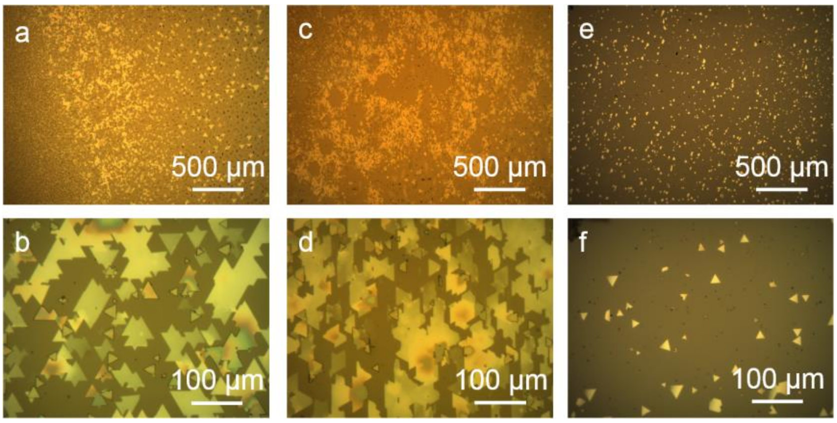

Figure S2. Characterization of 2D Fe_3O_4 nanosheets. a-d) Optical images of Fe_3O_4 nanosheets grown on *a*-face Al_2O_3 substrates. e,f) Optical images of Fe_3O_4 nanosheets grown on *c*-face Al_2O_3 substrate.

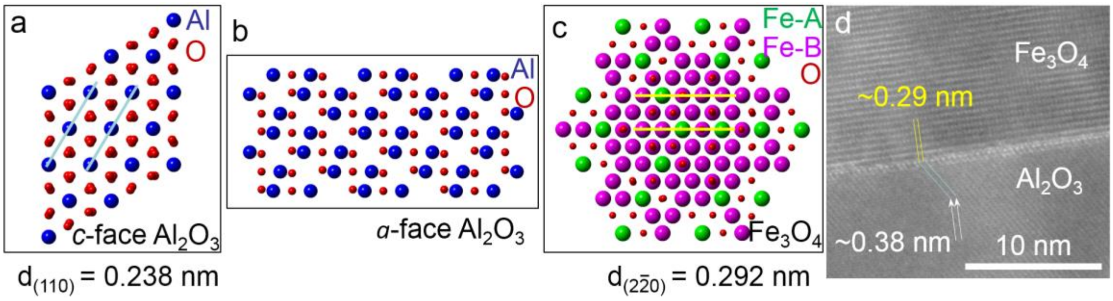

Figure S3. Schematic crystal structure of Fe_3O_4 and substrates. a,b) Atomic models illustrating the arrangement of Al and O atoms for c-face Al_2O_3 and a-face Al_2O_3, respectively. c) Atomic models illustrating the arrangement of Fe-A site, Fe-B site, and O atoms for Fe_3O_4 nanosheet. d) The TEM images of the cross-sectional samples show a sharp grain boundary between Fe_3O_4 and *c*-face Al_2O_3, exhibiting a specific crystallographic orientation relationship. The lattice spacing between them is ~0.29 nm and ~0.38 nm, respectively. Consequently, the calculated lattice mismatch ε between Fe_3O_4 and *c*-face Al_2O_3 is -23.7%, employing the equation of $\varepsilon = (a-b)/b$, where a and b represent the corresponding lattice spacings of the deposited crystalline layer and crystalline substrate, respectively.[3, 4]

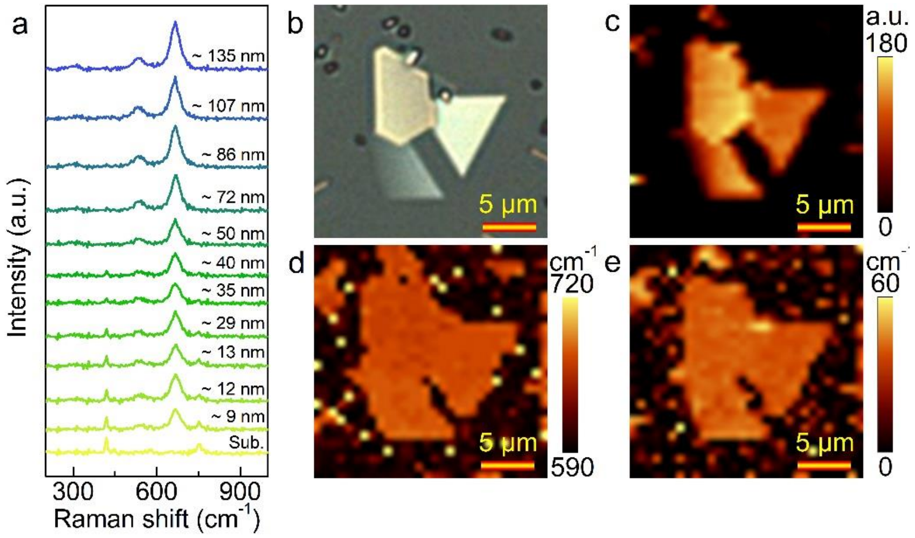

Figure S4. Characterization of Fe_3O_4 nanosheets with different thickness. a) Raman spectra of the Fe_3O_4 nanosheets with different thickness (in nanometers). b–e) Optical microscopy (OM) image (b) and the corresponding intensity (c), position (d), and width (e) mapping images of the Raman peak at ~666.4 cm^{-1} for three 2D Fe_3O_4 nanosheets.

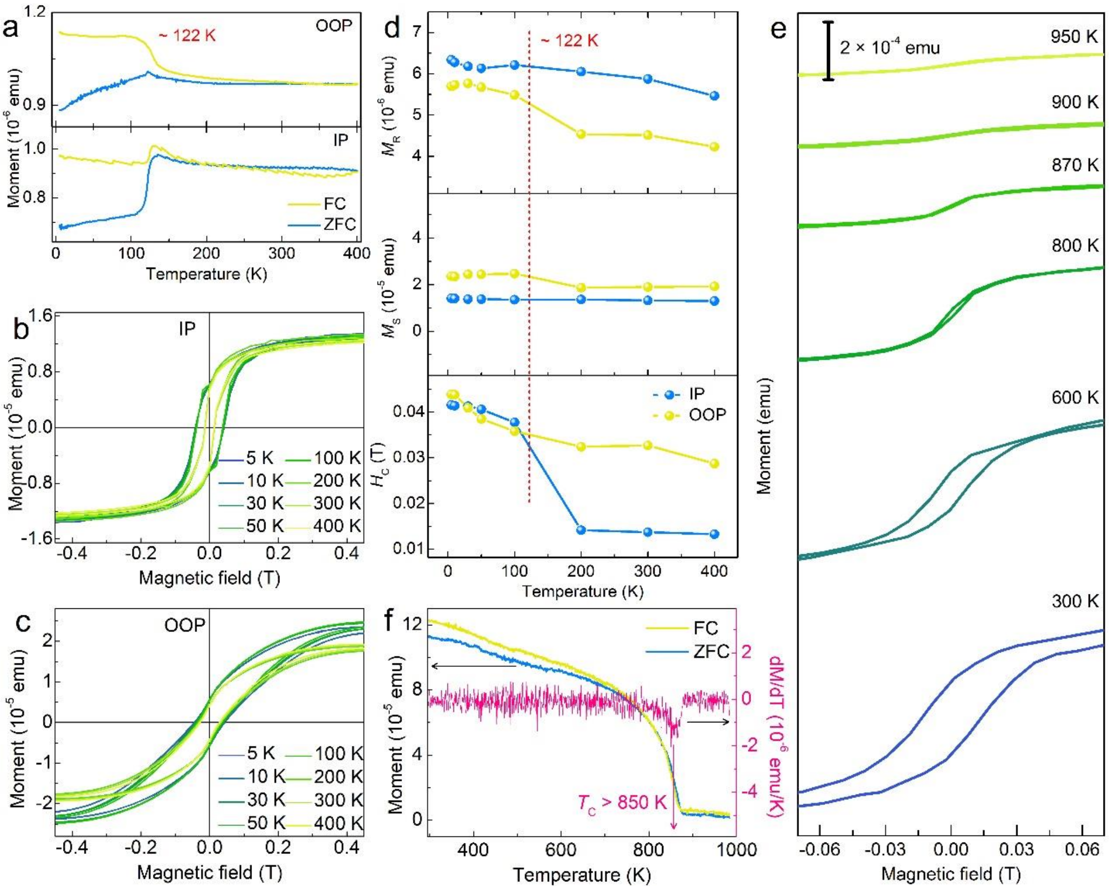

Figure S5. Magnetic properties of the Fe_3O_4 nanosheets. a) Temperature dependence of the zero-field-cooling (ZFC) and field-cooling (FC) magnetization for the Fe_3O_4 nanosheets under out-of-plane (OOP) and in-plane (IP) magnetic fields. b,c) Magnetic hysteresis (M-H) loop of the nanosheets at various temperatures under magnetic fields along the IP (b) and OOP (c) direction. d) Temperature-dependent remanent magnetization (M_R, top), saturation magnetization (M_S, center), and coercivity (H_C, bottom) obtained under IP and OOP magnetic fields. e) Magnetic moment as a function of the IP magnetic field measured at various temperatures. f) ZFC and FC magnetization and dM/dT curves for the Fe_3O_4 nanosheets under an IP magnetic field.

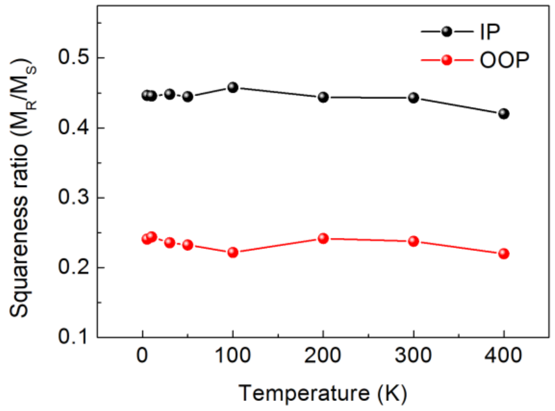

Figure S6. The squareness (M_R/M_S) versus temperature of Fe_3O_4 nanosheets under in-plane and out-of-plane magnetic fields, respectively.

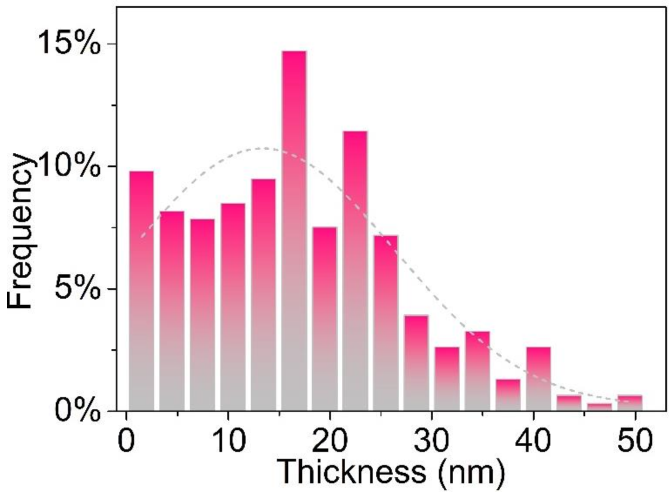

Figure S7. The thickness-size distribution of Fe_3O_4 nanosheets.

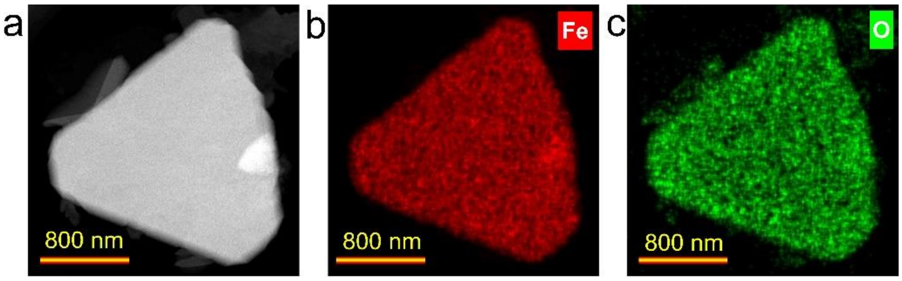

Figure S8. Structural and chemical analysis of a triangular nanosheet. a-c) HAADF STEM image (a) and the corresponding element distribution maps of Fe (b) and O (c) of a triangular Fe_3O_4 nanosheet.

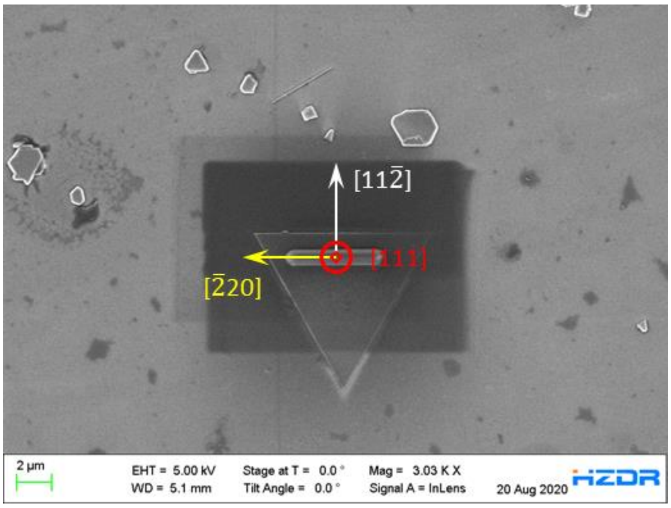

Figure S9. TEM sample preparation. Top-view SEM image of a triangular Fe_3O_4 nanosheet on Al_2O_3 taken during the FIB-based cross-sectional TEM lamella preparation. To prevent charging, the whole sample was coated with an Au film of ~10 nm thickness. To cover the sample surface at the position where the lamella is extracted, an additional rectangular C-based bar with a lateral size of about 1 µm × 5 µm was deposited. It should be noted that the dark-grey rectangular region in the center of the image is due contamination build-up during image acquisition caused by electron-beam-induced decomposition of remaining hydrocarbons.

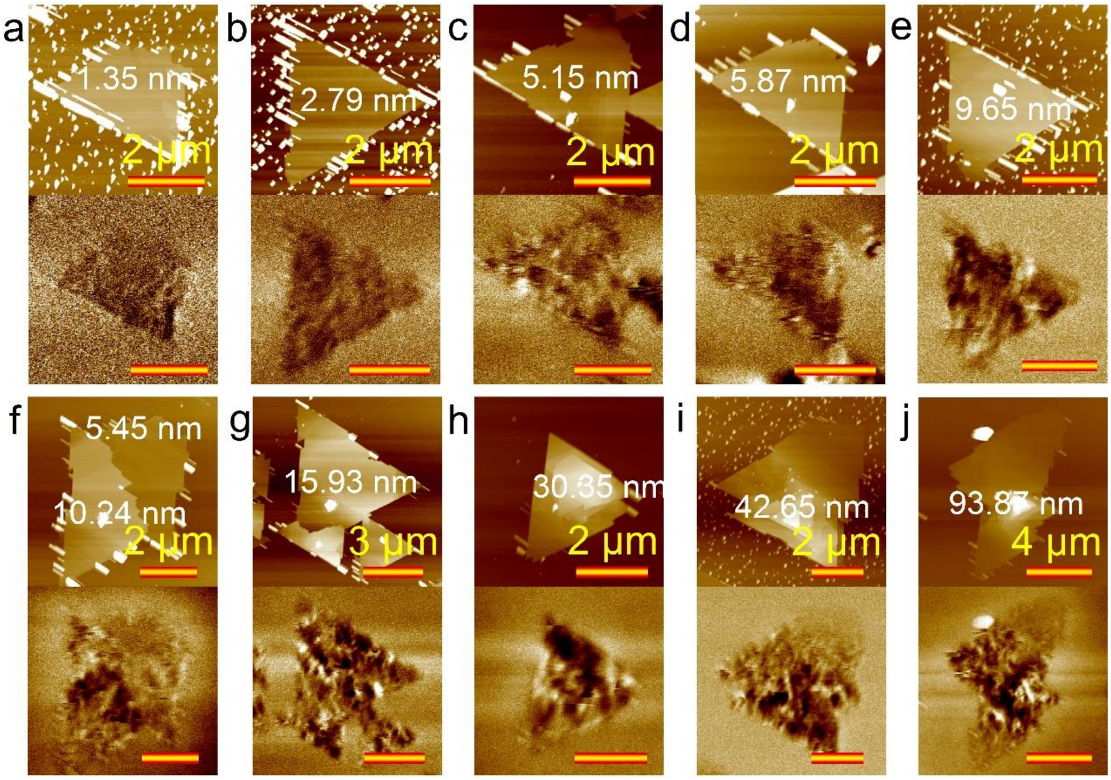

Figure S10. AFM and MFM characterization. a-j) AFM topography (top) and the corresponding in situ MFM images (bottom) for Fe_3O_4 nanosheets with different thickness. The thickness of the nanosheets is indicated by the numbers in the AFM images.

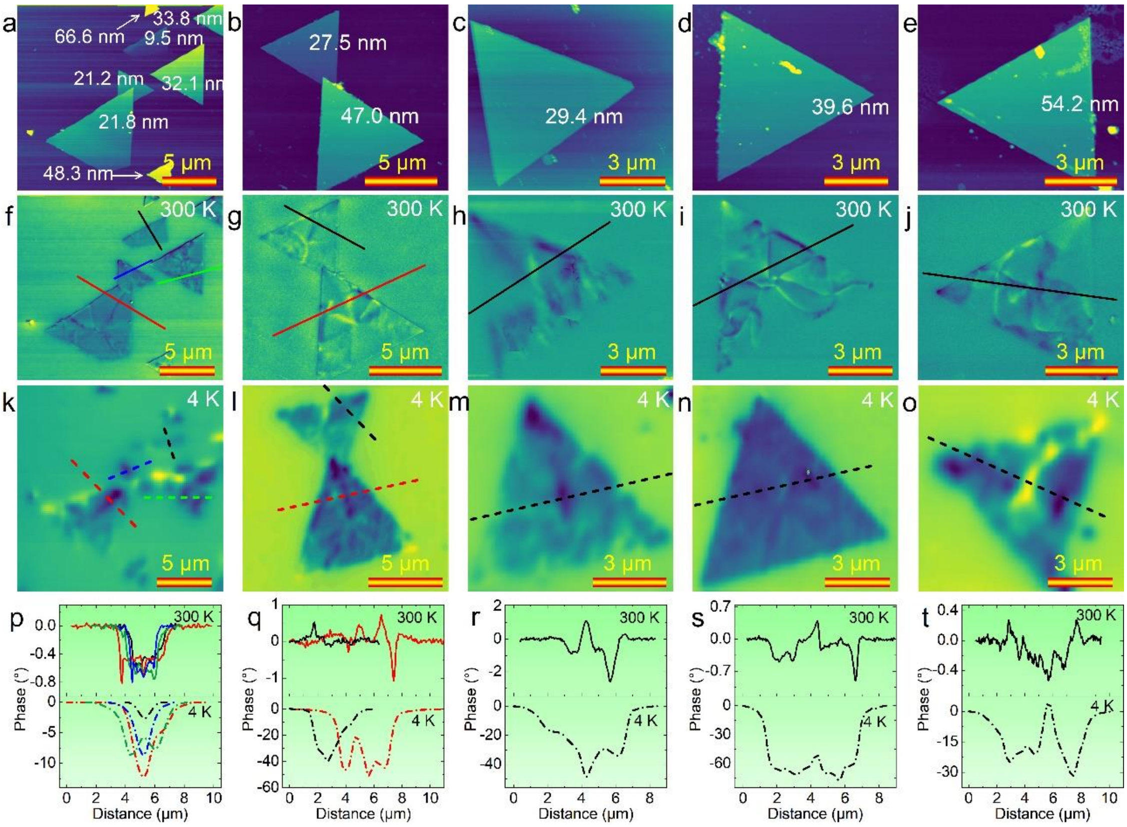

Figure S11. Temperature-dependent domain characterization. a–o) AFM topography (a-e) and the corresponding in-situ MFM images (f-o) for Fe_3O_4 nanosheets with different thickness measured at 300 K (f-j) and 4 K (k-o). The thickness of the nanosheets is indicated by the numbers in the AFM images. p–t) MFM phase angle line profiles measured at 300 K (top) and 4 K (bottom) obtained along the marked lines in the respective MFM images in (f–o).

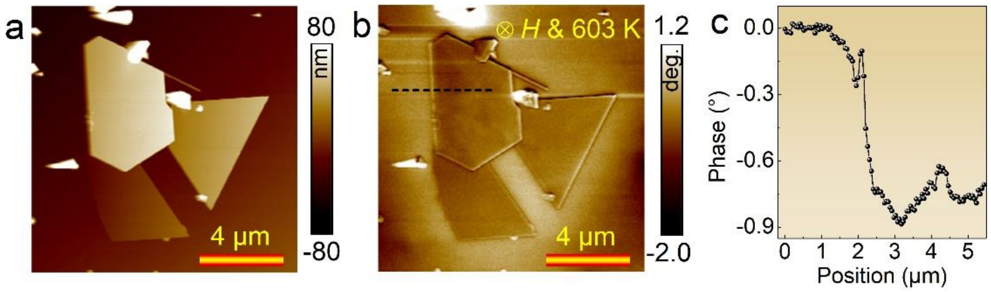

Figure S12. Characterization of the annealed samples. a,b) AFM (a) and MFM (b) images of the sample shown in Figure 3g after annealing at 603 K under an OOP magnetic field of 1 T. c) MFM phase angle acquired along the black dashed line marked in (b).

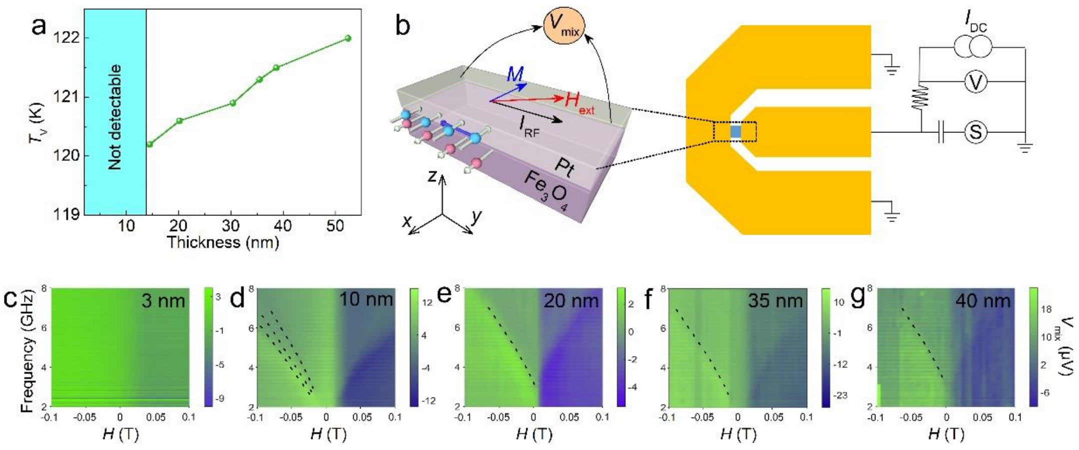

Figure S13. Thickness-dependent transition temperature and FMR characterization. a) Variation of transition temperature with the thickness of the Fe_3O_4 nanosheets. b) Schematic illustration of the Fe_3O_4/Pt (4 nm) ST-FMR measurement. c-g) FMR spectra mapping images for the Fe_3O_4 nanosheets with different thickness.

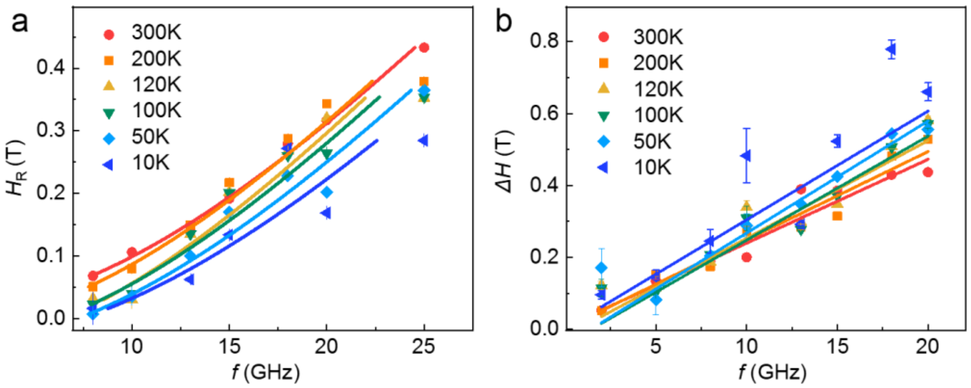

Figure S14. Frequency-dependent FMR behavior. a,b) Frequency dependence of the FMR resonance field (a) and the FMR resonance linewidth (b) at various temperatures.

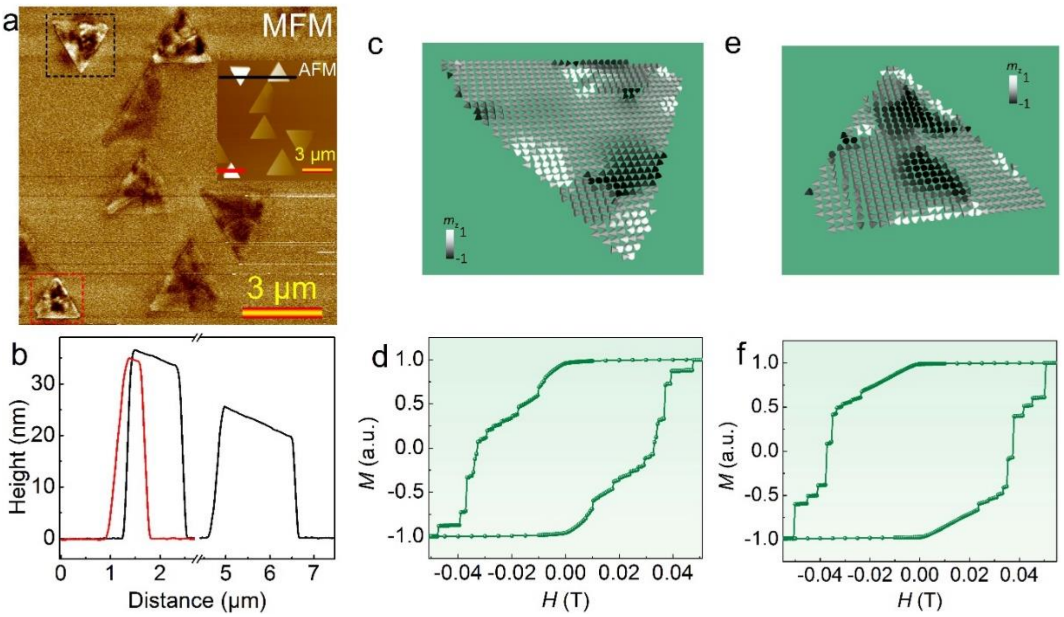

Figure S15. Micromagnetic simulation. a) MFM image of the materials used for micromagnetic simulation. The inset shows the AFM image of the nanosheets. b) Height profile of the selected nanosheets obtained along the black and the red line in the inset of (a). c,e) Simulation of the materials after relaxation under an internal field. The materials were chosen from the area marked by the black (c) and red (e) square in the MFM image in (a). d,f) Simulated hysteresis loops of the materials shown in the respective figures (c) and (e).

Reference

[1] F. Lan, R. Zhou, Z. Qian, Y. Chen, L. Xie, Chemical vapor deposition of ferrimagnetic Fe_3O_4 thin films, *Crystals* **2022**, *12*, 485.

[2] P. Wang, J. Ge, J. Luo, H. Wang, L. Song, Z. Li, J. Yang, Y. Wang, R. Du, W. Feng, J. Wang, J. He, J. Shi, Interisland-distance-mediated growth of centimeter-scale two-dimensional magnetic Fe_3O_4 arrays with unidirectional domain orientations, *Nano Lett.* **2023**, *23*, 1758.

[3] M. Liu, H. Ruan, L. Zhang, A. Moridi, Effects of misfit dislocation and film-thickness on the residual stresses in epitaxial thin film systems: Experimental analysis and modeling, *J. Mater. Res.* **2012**, *27*, 2737.

[4] C. Tan, J. Chen, X.-J. Wu, H. Zhang, Epitaxial growth of hybrid nanostructures, *Nat. Rev. Mater.* **2018**, *3*, 17089.
